# Assessment of Aging and Disability Concentration in a Master’s Program

**DOI:** 10.1093/geroni/igaa057.037

**Published:** 2020-12-16

**Authors:** Kristine Mulhorn

**Affiliations:** Drexel University, Philadelphia, Pennsylvania, United States

## Abstract

A concentration in aging and disability is an alternative concentration in our Master of Health Administration (MHA) program. Students to take both of their electives in this area as well as completing their residency in a post-acute care setting. To ensure students meet the learning objectives, the following assessment plan. First, a curricular map of the areas”” of competency within our program are outlined and then the content and areas of assessment are considered based on whether the competencies are “Introduced”, “Reinforced,” and “Mastered.” Next, the key assignments in each course within the concentration were aligned with the Program Objectives for the MHA Program. Another assessment strategy involves the competencies of the on-campus residency. A comparison of the competencies of the residency for those students who conducted their residency in the acute care setting to those competencies of the residency for the students who completed their residency in a post-acute setting. A final method for assessment is a review of the evaluations and the final paper for the residency. The residency is a capstone project in which students conduct analysis based on the operations management factors within the facility.

